# Mean Amplitude of Diurnal Variation of Sub-Foveal Choroidal Thickness among Adults with Healthy Eyes Visiting Outpatient Department of a Tertiary Eye Care Centre

**DOI:** 10.31729/jnma.8436

**Published:** 2024-02-29

**Authors:** Raju Kaiti, Priya Bajgai, Ranjila Shyangbo

**Affiliations:** 1 Department of Optometry, Nepal Eye Hospital, Tripureshwor, Kathmandu, Nepal; 2 Department of Ophthalmology, Nepal Eye Hospital, Tripureshwor, Kathmandu, Nepal; 3 Department of Optometry, National Academy of Medical Sciences, Mahaboudha, Kathmandu, Nepal

**Keywords:** *choroid*, *circadian rhythm*, *cross-sectional study*, *Ophthalmology*

## Abstract

**Introduction:**

The choroid, particularly the choroidal vasculature, plays an important role in the pathophysiology of various ocular diseases. Diurnal variation in the thickness of the choroid has been reported in previous studies. This study aimed to find the mean amplitude of diurnal variation of sub-foveal choroidal thickness among adults with healthy eyes visiting the outpatient Department of a tertiary eye care centre.

**Methods:**

A descriptive cross-sectional study was conducted among adults with healthy eyes presenting to the outpatient Department of a tertiary eye care centre from 1 February 2023 to 3 June 2023 after obtaining ethical approval from the Institutional Review Committee. After a comprehensive medical eye examination, sub-foveal choroidal thickness measurement was performed using enhanced depth imaging optical coherence tomography at the baseline and subsequent interval afterwards. A convenience sampling method was used. The point estimate was calculated at a 95% Confidence Interval.

**Results:**

Among 60 eyes, the mean amplitude of the diurnal variation of subfoveal choroidal thickness was 35.16±14.08 microns (31.59-38.72, 95% Confidence Interval). The mean amplitude for the right eye was 36.30±14.08 microns, and for the left eye, it was 34.23±14.08 microns.

**Conclusions:**

The amplitude of diurnal variation of subfoveal choroidal thickness from this study was found to be similar to other studies done in international settings.

## INTRODUCTION

The choroid, a vital vascular layer within the eye, plays a pivotal role in ocular blood supply. Traditionally, invasive procedures like indocyanine green and fluorescein angiography were employed to examine choroidal structures and vasculature. However, recent advancements in imaging technology, such as enhanced depth imaging-optical coherence tomography (EDI-OCT), have enabled non-invasive exploration of choroidal parameters. Enhanced depth imaging, optimized OCT acquisition parameters to visualize the full choroidal thickness, fostering easier choroidal image acquisition and analysis via longer wavelength imaging.^1^

The choroid, especially its vasculature, significantly impacts the pathophysiology of various ocular diseases, including myopia, central serous chorioretinopathy, polypoidal choroidal vasculopathy, age-related macular degeneration, diabetic retinopathy, and Vogt-Koyanagi-Harada disease.^2-4^ Choroidal blood flow lacks autoregulation and responds to systemic and ocular factors, like blood pressure and intraocular pressure.^5^ Nevertheless, studies on normative choroidal physiology in Nepal are limited.

This study aimed to find the mean amplitude of diurnal variation of sub-foveal choroidal thickness among adults with healthy eyes visiting the outpatient Department of a tertiary eye care centre.

## METHODS

A descriptive cross-sectional study was conducted among healthy adults presenting to the outpatient department of Nepal Eye Hospital, Tripureswor, Kathmandu, Nepal from 1 February 2023 to 3 Jun 2023. Ethical approval was taken from the Institutional Review Committe of the National Academy of Medical Sciences (Reference number: 502/2079/80). Adults with healthy eyes between the age group 20-30 years and absence of ocular and systemic disease and Best Corrected Visual Acuity (BCVA) of 0.00 Log MAR were included in the study. Adults with a history of prior ocular surgeries, IOP >21 mm of Hg and OCT images in which the choroido-scleral interface was not identified were excluded from the study. A convenience sampling method was used. The sample size was calculated by using the following formula: a ^2^


$$\begin{array}{ll} \text{n} & ={\text{Z}}^{2}\times \frac{\sigma^{\text{2}}}{{\text{e}}^{\text{2}}}\\ & =1.96^{2}\times \frac{14.8^{2}}{4^{2}}\\ & =53 \end{array}$$

Where,

n = minimum required sample size
Z = 1.96 at 95% Confidence Interval (CI)
SD = standard deviation taken from previous study^5^
e = margin of error

The calculated sample was 53. However, 60 eyes of 30 healthy adults were enrolled in this study.

Before enrollment, all participants underwent a thorough eye examination. Systemic assessments included blood pressure and body weight measurements, while ocular parameters recorded encompassed mean spherical refractive status, intraocular pressure (IOP), and axial length readings. The mean spherical error (MSE) is computed as the sum of the spherical power and half the cylinder power. The mean MSE value observed may be negative attributed to a higher number of patients with of myopia and emmetropia compared to hyperopia. These measurements were conducted before OCT assessments. OCT imaging was performed using a multimodal imaging device (Spectralis OCT; Heidelberg Engineering, Heidelberg, Germany) with a standardized scanning protocol. For each eye, a horizontal raster pattern scan (10 × 10 degrees) centred on the fovea, with tracking enabled, was carried out. This involved averaging 25 frames to enhance image quality. The enhanced depth imaging (EDI) technique, with the zero delay line oriented towards the choroidal side, was employed to optimize choroidal sensitivity and visualize the entire choroidal thickness. Sub-foveal choroidal thickness (SFCT) measurements were manually obtained using the calliper tool within the built-in software (Heidelberg Eye Explorer) on the OCT machine. This was done by drawing a line perpendicularly from the hyperreflective line believed to represent the retinal pigment epithelium (RPE) to the choroid-scleral junction.^1^ Choroidal thickness measurements were taken between 9 am and 3 pm, with the first OCT scan (9 am) recorded as the baseline and subsequent scans at 2-hour intervals until 3 pm.^5^ All scans were performed with the participants in a sitting position to minimize posture-related effects on choroidal thickness.

Data were entered and analyzed using IBM SPSS Statistics version 26.0. The point estimate was calculated at a 95% CI.

## RESULTS

**Figure 1:**
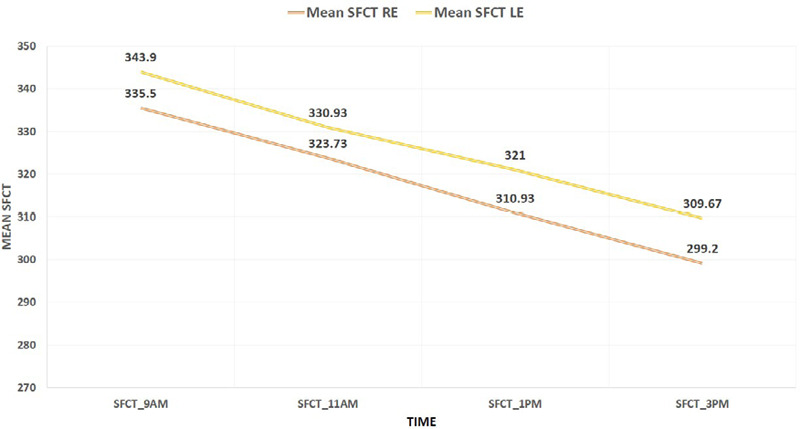
Change in mean SFCT at different time points (n= 60).

The mean amplitude of the diurnal variation of SFCT was 35.16±14.08 microns (31.59-38.72, 95% CI). Specifically, the mean amplitude for the right eye (RE) was 36.30±14.08 microns, and for the left eye (LE), it was 34.23±14.08 microns. The mean SFCT was maximum at 9 am and minimum at 3 pm for all eyes (Figure 1). The largest fluctuation in SFCT was 66 microns, and the smallest fluctuation in SFCT was 15 microns. The baseline SFCT of the right eye was 335.0±28.34 microns, and that of the left eye was 343.90±29.77 microns, with a mean between the two eyes of 339.70±29.13 microns.

The mean age of the subjects was 22.10± 1.98 years. The mean body weight of the subject was 50.67±7.86 kg. The baseline recorded systolic and diastolic blood pressure was 112±7.14 mm of Hg and 74±7.23 mm of Hg respectively (Table 1).

**Table 1:** Ocular data of enrolled adults (n = 60).

Characteristics	Right eye (Mean±SD)	Left eye (Mean±SD)
Mean spherical error	-0.57±1.18	-0.52±0.98
Mean axial length	23.07±0.97	23.06±0.94
Mean IOP	14.33±3.30	14.63±2.80

## DISCUSSION

In this study, the mean amplitude of diurnal variation of subfoveal choroidal thickness was 35.16±14.08 microns, which is similar to a study reported in the Indian population (35.91±14.8 μim).^5^ Few studies have also reported a slightly higher amplitude than this study, 40 microns and 44 microns respectively.^6,7^ In the Japanese population, a mean diurnal fluctuation of about 33 microns in SFCT has been reported.^8^ The variation in amplitude could be linked to differences in the study age group, as both choroidal thickness and the amplitude of diurnal variation of SFCT are known to vary with age. Additionally, both studies had similar axial lengths to this study (23.9±1.33 mm and 23.83±1.38 versus (23.07±0.97). Because choroidal vessels have poor autoregulation, changes in perfusion pressure have a direct effect on blood flow.^8^

Diurnal variation is well documented in human eyes for parameters such as intraocular pressure (IOP), axial length (AL), and SFCT. In this study, we looked at the diurnal variation of sub-foveal choroidal thickness in healthy Nepalese subjects and discovered a typical diurnal variation pattern of SFCT that was highest in the morning, then began to drop, and reached a minimum value as the sunset.^6-9^ This is consistent with most studies which commonly show thicker sub-foveal choroidal thickness in the morning and thinner in the evening, correlating with daily fluctuations in blood pressure. However, contrasting findings have also been reported where there was an increase in SFCT as the day progressed.^10,11^

The baseline SFCT in this study was 339.70±29.13 microns, surpassing the previously reported SFCT in two recent Nepalese studies (308.89±68.87 μm and 310.31 ±75.70 μm, respectively).^12,13^ The baseline SFCT was also higher than that reported in the Indian population (307±79 μm), Spanish population (301.89±80.53 μm), and Chinese population (328.8 μm).^14-16^ This discrepancy may be attributed to the variation in the age group enrolled; our study exclusively included healthy adults aged between 20-30 years, whereas the aforementioned studies aimed to assess mean SFCT across a broader age range, from pediatric to geriatric populations.

There are a few limitations in our study. Firstly, the sample size was relatively small, thus the generalization of findings from this study to the general population may not be entirely true. Diurnal variation was only measured from morning to early evening but not through the evening and night, making it difficult to point out the true maximum and minimum values of SFCT. Furthermore, SFCT was measured manually with an in-built calibre, which may have introduced measurement bias. Choroidal thickness exhibits differences from centre to periphery, but in this study, only SFCT has been measured. Further studies with a larger sample size, and measurement across extended choroidal intervals will be necessary to endorse these results.

## CONCLUSIONS

The mean amplitude of diurnal variation of subfoveal choroidal thickness from this study is similar to the amplitude reported from other international studies. It is imperative to evaluate these fluctuations of choroidal thickness to allow comparison of this physiological phenomenon in various disease processes involving the retina and choroid, which will further aid in a better understanding of their pathophysiology.
